# Treatment compliance among adult cervical cancer patients receiving care at Uganda cancer institute, Uganda: a retrospective data review

**DOI:** 10.1186/s12885-023-11145-1

**Published:** 2023-07-05

**Authors:** Josephine Irene Najjemba, Regina Ndagire, Pius Mulamira, Solomon Kibudde, Catherine Nassozi Lwanira

**Affiliations:** 1 School of Nursing and Midwifery, Clarke International University, PO Box 7782, Kampala, Uganda; 2 Uganda Cancer Institute, PO Box 3935, Kampala, Uganda

**Keywords:** Cervical cancer, Treatment compliance, Uganda

## Abstract

**Background:**

Cervical cancer is one of the most common cancers and a major cause of morbidity among women globally. Chemoradiation therapy is the preferred standard treatment for women with stage IB to IVA. However, the benefits of this treatment can only be achieved if patients adhere to the treatment guidelines. In this study, the proportion of compliance or adherence to chemo-radiation treatment among cervical cancer patients at Uganda Cancer Institute (UCI) was determined.

**Methods:**

This was a cross-sectional study that reviewed data retrospectively for 196 cervical cancer patients who were prescribed to chemo-radiation therapy at UCI between November 2020 to May 2021, having been diagnosed with disease stage IB to IVA. Patient data and information on treatment uptake was obtained by review of the patient’s medical records. Treatment compliance was determined by calculating the number of participants who completed the prescribed treatment (definitive pelvic concurrent chemoradiation to 50 Gy external beam radiotherapy with weekly concurrent cisplatin followed by intracavitary brachytherapy 24 Gy in 3 fractions at 8 Gy once a week over 3 weeks). Associations between patient factors and treatment adherence were determined using logistic regression analysis. In all statistical tests, a *P*- value of < 0.05 was considered as significant.

**Results:**

The proportion of patients who were administered with external beam radiation (EBRT), chemotherapy and brachytherapy were 82.6%, 52.04% and 66.2% respectively. However, only 23 of 196 patients (11.7%) were found to have adhered to the treatment plan by completion of all definitive pelvic concurrent chemoradiation to 50 Gy external beam radiotherapy (5 weeks) with weekly concurrent cisplatin (5 cycles) followed by intracavitary brachytherapy 24 Gy in 3 fractions at 8 Gy once a week over 3 weeks (3 sessions). There were no significant associations between patient factors and treatment adherence after multivariable analysis.

**Conclusions:**

Treatment compliance was found in only 12% of the cohort participants. No association of patient factors with treatment compliance was found. Additional studies on treatment adherence with larger sample sizes are needed to confirm the associations.

## Background

Cervical cancer continues to be a major cause of morbidity and mortality among women globally, accounting for over 604,000 new cancer cases and 342,000 deaths in 2020 [1]. The greater burden of the disease is reported in low- and middle-income countries, where it is the third most common cancer among women [1]. Uganda is among the countries with the highest incidence rates of cervical cancer, with over 20.5% of new cases reported in 2020 [2]. The incidence rate in Uganda is estimated at 52.6 per 100,000 population and this is projected to increase to 66.1 per 100,000 population by 2030 [3]. According to Kampala Cancer Registry, more than 80% of women with cervical cancer are referred to Uganda Cancer Institute (UCI) with advanced disease, usually at stage III or higher [4], yet the survival rates from cervical cancer remain considerably low [2].

Chemoradiation therapy is the recommended standard treatment for women with stage IB to IVA disease [5, 6], because it is known to improve the quality of life and to increase patient survival [6, 7]. However, the beneficial effects of chemoradiation therapy can only be realized if patients adhere to treatment [8]. Good compliance or adherence implies that the patient follows recommendations by the treatment provider on timing, dosage, and frequency of medication [9], however, this remains a significant challenge for many people living with cancer in resource-limited settings [10–14]. Several studies done previously in the sub-Saharan region have reported considerable treatment non-compliance rates among cervical cancer patients [13, 15, 16], with patient loss to follow-up of up to 15% or greater [17, 18]. In a more recent study done in Zimbabwe’s Parirenyatwa hospital, radiotherapy uptake was reported in about 86% of the patients, with only 38% receiving concurrent chemotherapy [13]. Many of these studies however, have been done in populations outside Uganda. There is still limited information regarding chemoradiation treatment adherence among cervical cancer patients in Uganda.

According to anecdotal reports from UCI, over 75% of cervical cancer patients at UCI are indicated for chemo-radiation therapy but more than half of the patients are found to not adhere to the treatment plan. Poor adherence to cancer medication has been associated with decreased survival [7], higher recurrence/treatment failure rates [19], and increase of health care costs [20]. Without adequate investment in cervical cancer control and increased adherence to chemoradiation, the rates of cervical cancer are only expected to rise [21]. However, in Uganda the burden of chemoradiation non-adherence among cervical cancer patients is not documented. In this study, the proportion of compliance or adherence to chemo-radiation treatment among cervical cancer patients at UCI was determined. Understanding the rate of treatment compliance is important in providing baseline data of value in cervical cancer interventions.

## Methods

### Study design and setting

This was a cross-sectional study that reviewed data retrospectively from a cohort of cervical cancer patients receiving care at UCI in Uganda. UCI is an autonomous, specialized public medical care facility owned by Uganda’s Ministry of Health, located approximately 5 Km North East of the central business district of Kampala, along upper Mulago hill road. It is the home of East Africa’s center of excellence for oncology serving a combined population of 170 million people.

### Patient enrolment

The study cohort was recruited from a population of cervical cancer patients who were on chemo-radiation therapy at UCI between November 2020 to May 2021, having been diagnosed with disease stage IB2 to IVA according to the National Comprehensive Cancer Network (NCCN) guidelines for cervical cancer [22]. Treatment of cervical cancer followed standard guidelines, which included pelvic external beam radiation (EBRT) + concurrent platinum-containing chemotherapy, followed by intracavitary brachytherapy [22]. EBRT dose was 50 Gy in 25 fractions, given in 2 Gy per fraction daily for 5 weeks along with weekly cisplatin at 40 mg/m^2^ for 5 weeks, after assessing for creatinine clearance and other blood parameters such as complete hemogram, random blood sugar, and liver function tests. After completion of EBRT, three fractions of weekly high dose rate (HDR) brachytherapy were given, starting after a gap of one week at a dose of 8 Gy per fraction. The total duration of completion of treatment with concurrent chemoradiation and brachytherapy was kept around 56 days (8 weeks) [6, 23]. Eligible participants were duly expected to have completed all the prescribed chemo-radiation therapy cycles plus the additional intracavitary brachytherapy treatment.

### Sample size determination

The sample size was estimated using Yamane’s (1967:886) formular [24] given by:

$$\mathrm n=\mathrm N/\left(1+\mathrm{Ne}^2\right)$$

where: n is the minimum sample size required for the study.

N is the size of the population under study.

e is the margin of error (5%).

According to the UCI medical records, approximately 320 cervical cancer patients were prescribed to chemo-radiation therapy between October 2020-May 2021.

Hence, considering an approximate population size of 320 cervical cancer patients, *n* = 320/1+(320 × 0.05^2^) = 178. Then, the corrected sample size with a 10% contingency for incomplete medical records provided a final sample size of 196 cervical cancer patients.

### Data collection

Patients were examined for cervix cancer by attending gynecology–oncologist physicians at UCI. Patients who were diagnosed with disease stage IB to IVA were prescribed to chemoradiation treatment following standard treatment guidelines [22]. Patient information was obtained from the medical files of patients who were duly expected to have completed all the prescribed chemo-radiation therapy cycles plus the additional intracavitary brachytherapy treatment. Out of the total 218 medical files of eligible participants, 196 patient files were selected by simple random sampling and all participants provided informed consent. Baseline data including age, residence, marital status, occupation, religion, kinship, medical history, surgical history and type of surgery done, presence of co-morbidities, disease stage at presentation, history of cancer in the family and treatment cycle was extracted from the patient files using data abstraction forms. A team of well-trained health workers and researchers collaborated to cross-check patient data and verify the completeness and accuracy of the data. Patient files with incomplete medical records were excluded. Treatment compliance was determined by having completed all the scheduled chemo-radiation (5 weeks of EBRT and 5 cycles of chemotherapy) and 3 insertions of brachytherapy as per the chart and completion of the treatment plan as prescribed by the gynecology–oncologist.

### Data management and analysis

Data were entered and cleaned using EpiData version 4.6.0.2. Descriptive statistics, bivariate and multivariate analysis were carried out using STATA 14.0 statistical software (STATA, College Station, Texas). Means and standard deviations (SD) were calculated for normally distributed continuous data, while proportions and frequencies were used for categorical variables. The proportion of patients who were adherent to treatment was determined by calculating the number of participants who completed the prescription (5 weeks of EBRT, 5 cycles of chemotherapy, and 3 insertions of brachytherapy) divided by the study sample size.

The association between patient factors and treatment adherence was assessed using bivariate and multivariate analysis. Initial bivariate analysis identified significant variables (*P*-values < 0.2) that were included in the final logistic regression analysis to determine the extent to which treatment compliance was affected by patient factors. Adjusted odds ratios (AORs) and their corresponding 95% confidence intervals (Cl) were calculated. All statistical tests were two-tailed and *P*-values < 0.05 were regarded as significant.

## Results

### Patient characteristics

A total of 196 patient files were reviewed. The mean patient age was 51.6 ± 13.0 years. Slightly below half of the study participants (46.9%) were married, the majority (61.3%) were in informal employment, 43.5% were Catholics, and almost all patients (99.5%) had a next of kin. At presentation, above half of the patients (54.9%) were in disease stage IIIA-IIIB, but 68.2% of the respondents had patient investigations completed. Patients were examined for other co-morbidities at presentation. The majority (71.9%) reported no underlying medical illness, while 67 of 194 (34.5%) were known to be HIV positive. Many participants (73.7%) reported no previous surgical operation and only 19 of 192 (9.9%) reported having a history of cervical cancer in the family. Details of the patient demographic and clinical characteristics are given in Table 1.

**Table 1:** Patient demographic and clinical characteristics

Patient characteristic	Category	Frequency (n)	Percentage (%)
Age [mean (SD)]	51.6 (13.0)		
Marital status (*n* = 192)	Married	90	46.88
	Single	64	33.33
	Widow	22	11.46
	Divorced/separated	16	8.33
Occupation (*n* = 194)	Formal employment	13	6.70
	Informal	119	61.34
	Self employed	62	31.96
Religion (*n* = 193)	Catholic	84	43.52
	Anglican	71	36.79
	Moslem	29	15.03
	Others (B/A, SDA& Ortho)^a^	9	4.66
Had next of kin (*n* = 196)	Yes	195	99.49
	No	1	0.51
Relationship with next of kin (*n* = 196)	Mother/father	9	4.59
	Daughter/son	92	46.94
	Brother/sister	25	12.76
	Husband	61	31.12
	Other relative	9	4.59
Medical history (*n* = 192)	Yes	54	28.13
	No	138	71.88
Surgical history (*n* = 190)	Yes	50	26.32
	No	140	73.68
Disease stage (*n* = 193)	Stage IB2- IB3	18	9.33
	Stage IIA-IIB	61	31.61
	Stage IIIA-IIIB	106	54.92
	Stage IVA	8	4.15
HIV status (*n* = 194)	Yes	67	34.54
	No	127	65.46
Family history of Cervical cancer (*n* = 192)	Yes	19	9.90
	No	173	90.10
Completed investigations (*n* = 192)	Yes	131	68.23
	No	61	31.77

^a^ *B/A* Born again Christian, *SDA *Seventh-day Adventist, *Ortho *Orthodox

### Uptake of chemo-radiation therapy in the study population

In this study, 147 of 178 patients (82.6%) were initiated on EBRT, and only slightly above half (102 of 196; 52.04%) received concurrent chemotherapy. The proportion of patients who received the additional brachytherapy was 66.2%. Details of the uptake of chemoradiation treatment are given in Table 2.

**Table 2:** Number of patients who received each of EBRT, chemotherapy and brachytherapy

Treatment received		Frequency (n)	Percentage (%)
EBRT (*n* = 178)^a^	Yes	147	82.58
	No	31	17.42
Chemotherapy (*n* = 196)	Yes	102	52.04
	No	94	47.96
Brachytherapy (*n* = 192)	Yes	127	66.15
	No	65	33.85

^a^ *EBRT *External beam radiation

**Fig. 1:**
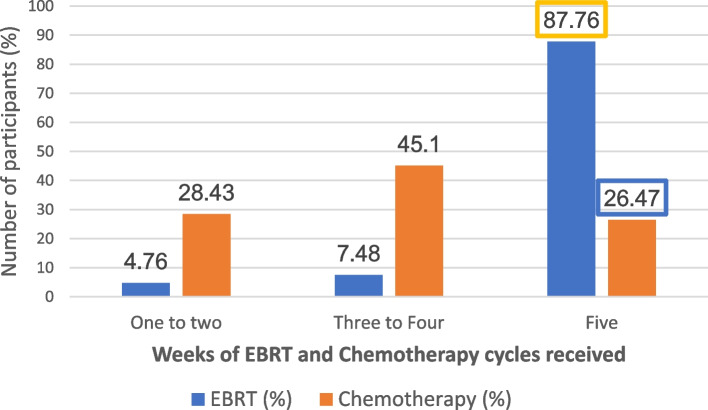
Percentage of patients administered with EBRT and concurrent chemotherapy. 88% of the patients completed all 5 weeks of EBRT, while 26.5% completed all 5 cycles of chemotherapy

**Fig. 2:**
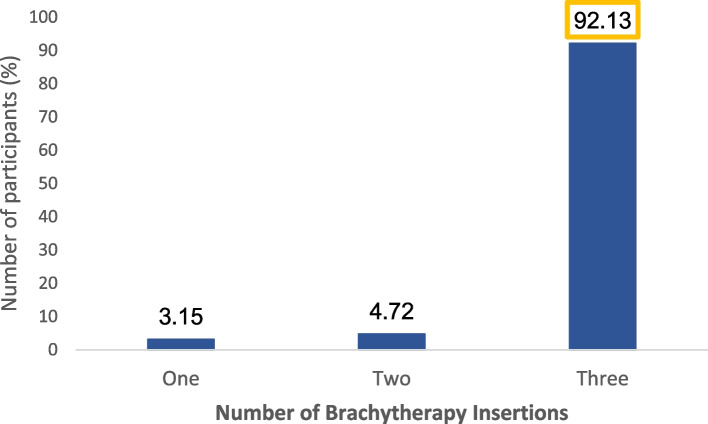
Percentage of patients administered with intracavitary brachytherapy. 92% of the study participants completed all 3 insertions of brachytherapy

There was no consistency in uptake for all prescribed chemoradiation schedule. The proportion of patients who completed all five weeks of EBRT was 87.8%. However, only 27 of the 102 patients (26.5%) completed the five cycles of chemotherapy. The number of weeks of EBRT, and cycles of chemotherapy received are shown in Fig. 1. Completion of treatment was highest for brachytherapy with 117 of the 127 patients (92.1%) having completed all the three insertions, as shown in Fig. 2.

### Adherence to chemo-radiation treatment in the study population

**Fig. 3:**
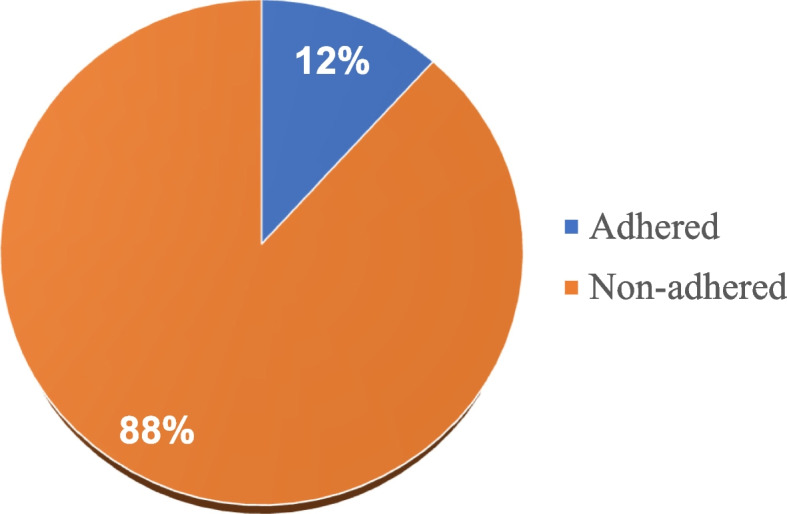
Adherence to chemo-radiation treatment. Only 12% of the patients completed all 5 weeks of EBRT, 5 cycles of chemotherapy and 3 insertions of brachytherapy as prescribed by the gynecology–oncologist

In this study, only 23 of 196 patients (11.7%) adhered to the treatment plan by completing of all the prescribed cycles (5 weeks of EBRT, 5 cycles of chemotherapy, and 3 insertions of brachytherapy). The highest number (88.3%) did not adhere to the prescribed chemo-radiation treatment (see Fig. 3).

### Relationship between patient factors and adherence to chemo-radiation treatment

The association between patient factors and adherence to chemo-radiation was determined using multivariate logistic regression analysis. An initial bivariate analysis (Table 3) identified the patient’s occupation (*p* = 0.030), disease stage (*p* = 0.073), distance (*p* = 0.109) and having missed a medical appointment (*p* < 0.001) to be significantly associated with the patient’s adherence to chemoradiation treatment. However, no single patient factor was found to be independently associated with the patient’s adherence to chemo-radiation treatment after multivariable analysis. Details of the multivariate analysis of patient factors and adherence to chemo-radiation treatment are given in Table 4.

**Table 3:** Bivariate analysis of patient factors and adherence to chemo-radiation treatment

		**Outcome**		**Bivariate**		
**Patient factor**	**Category**	**Adhered**		**Unadjusted OR**	**95%Cl**	***P*** **-value**
		No, n (%)	Yes, n (%)			
Age (years)	< 51	85(88.54)	11(11.46)	0.95	0.39 - 2.27	0.906
	≥ 51	88(88.0)	12(12.0)	Reference		
Marital status (*n*=192)	Married	81(90)	9(10)	Reference		
	Single	55(85.94)	9(14.06)	1.47	0.55 - 3.95	0.441
	Widow/Divorced/separated	35(92.11)	3(7.89)	0.77	0.20 - 3.02	0.710
Occupation (*n*= 194)	Formal employment	9(69.23)	4(30.77)	4.36	1.15 - 16.52	*0.030
	Informal	108(90.76)	11(9.24)	Reference		
	Self-employed	54(87.1)	8(12.9)	1.45	0.55 - 3.83	0.448
Religion (*n*=193)	Catholic	74(88.1)	10(11.9)	Reference		
	Anglican	64(90.14)	7(9.86)	0.81	0.29 - 2.25	0.685
	Moslem	25(86.21)	4(13.79)	1.18	0.34 - 4.11	0.790
	Others (B/A, SDA& Ortho)	8(88.89)	1(11.11)	0.93	0.10 - 8.19	0.944
Relationship with next of kin	Mother/father	9(100)	0(0)	1	-	-
	Daughter/son	83(90.22)	9(9.78)	Reference		
	Brother/sister	21(84.0)	4(16.0)	1.76	0.49 - 6.26	0.377
	Husband	53(86.89)	8(13.11)	1.39	0.51 - 3.83	0.521
	Other relative	7(77.78)	2(22.22)	2.63	0.47 - 14.64	0.241
Medical history (*n*= 192)	No	120(86.96)	18(13.04)	Reference		
	Yes	50(92.59)	4(7.41)	0.53	0.17 - 1.65	0.277
Surgical history (*n*= 190)	No	125(89.29)	15(10.71)	Reference		
	Yes	42(84.0)	8(16.0)	1.58	0.63 - 4.00	0.328
Disease stage (*n*=193)	Stage IAI- IB2	16(88.89)	2(11.11)	1.34	0.27 - 6.81	0.719
	Stage IIA-IIB	50(81.97)	11(18.03)	2.37	0.92 - 6.10	*0.073
	Stage IIIA-IIIB	97(91.51)	9(8.49)	Reference		
	Stage IVA-IVB	8(100)	0(0)	1	-	-
HIV status	No	110(86.61)	17(13.39)	Reference		
	Yes	61(91.04)	6(8.96)	0.63	0.24 - 1.70	0.367
Family history of Cervical cancer	No	154(89.02)	19(10.98)	Reference		
	Yes	15(78.95)	4(21.05)	2.16	0.73 - 5.05	0.209
Completed investigations	No	56(91.8)	5(8.2)	0.56	0.20 - 1.59	0.276
	Yes	113(86.26)	18(13.74)	Reference		
Distance (km)	1-5	8(72.73)	3(27.27)	3.23	0.77 - 13.48	*0.109
	6-10	25(86.21)	4(13.79)	1.37	0.47 - 3.70	0.593
	11-20	9(90.0)	1(10.0)	0.96	0.42 - 4.49	0.967
	>20	129(89.58)	15(10.42)	Reference		
Missed appointment	No	1(5.0)	19(95.0)	1051.33	104.10 -	*<0.001
	Yes	166(98.22)	3(1.78)	Reference	10617.2	
All investigations done at UCI	No	149(87.65)	21(12.35)	Reference		
	Yes	23(92.0)	2(8.0)	0.62	0.14 - 2.80	0.532

*OR* Odds ratio, *CI *Confidence interval

^*^Refers to significant *p*-value

**Table 4:** Multivariate analysis of patient factors and adherence to chemo-radiation treatment

		**Outcome**				
**Patient factor**	**Category**	**Adhered**		**Adjusted OR**	**95%Cl**	***P*** **-value**
		No, n (%)	Yes, n (%)			
Occupation (*n*= 194)	Formal employment	9(69.23)	4(30.77)	1.31	0.04 - 46.02	0.882
	Informal	108(90.76)	11(9.24)	Reference		
	Self-employed	54(87.1)	8(12.9)	0.48	0.03 - 6.72	0.586
Disease stage (*n*=193)	Stage IAI- IB2	16(88.89)	2(11.11)	3.43e-07	0 - -	1.000
	Stage IIA-IIB	50(81.97)	11(18.03)	2.16e-13	0 - -	1.000
	Stage IIIA-IIIB	97(91.51)	9(8.49)	Reference		
	Stage IVA-IVB	8(100)	0(0)	1	-	-
Distance (*n*=194)	1-5	8(72.73)	3(27.27)	1.36	0.02 - 78.91	0.881
	6-10	25(86.21)	4(13.79)	0.41	0.02 - 10.75	0.597
	11-20	9(90.0)	1(10.0)	3.34e-07	1.0	1.000
	>20	129(89.58)	15(10.42)	Reference		
Missed appointment (*n*=189)	No	1(5.0)	19(95.0)	1.52e+15	0 - -	1.000
	Yes	166(98.22)	3(1.78)	Reference		

## Discussion

Chemoradiation treatment non-compliance has been reported in studies done previously in low- and middle-income countries [13, 15, 16], however, most of these studies were done in populations outside Uganda. In the present study, the rate of compliance to chemoradiation treatment among cervical cancer patients at Uganda Cancer Institute was determined. This was a cross-sectional study that reviewed medical records retrospectively for cervical cancer patients that were prescribed to chemoradiation treatment. The study also benefits from the patient clinical data to determine associations between patient factors and treatment compliance.

In this study, only 12% of the patients were found to have adhered to the prescribed chemoradiation treatment plan. Majority of the patients who were initiated on treatment completed one or a combination of two treatment modalities; either radiotherapy (87.8%) or chemotherapy (26.5%) or brachytherapy (92.1%) or a combination of two, but not all the prescribed cycles of chemoradiation. This level of adherence is low when compared to that reported in previous studies in similar settings [15, 16] and to the adherence rates reported in a systematic review done elsewhere of 42–54% [7]. In the Kenyan and Ethiopian studies, adherence rates were 67.9% and 69.7% respectively [15, 16], which was attributed to the availability of better facilities and increased awarenesss of patients regarding medication adherence. Similarly, a previous systematic review reporting higher adherence rates [7] included studies done in California, USA where there are better health care services. Comparatively, the present study was done in Uganda where there is still inadequate infrastructure for cancer care. There are high costs associated with cancer treatment, treatment delays are reported largely due to inefficiencies within the health care system and the poor-economic status of the patients. Recent studies done at UCI confirm delays in the initiation and continuation of cancer treatment due to shortage of specialists, long waiting hours, patients requiring visits to outside facilities for staging investigations, prohibitive costs, poor navigation system and time wastage [14, 25]. Also notably, this study was carried out during November 2020 to May 2021, a period when the nation was on the road to recovery from the COVID − 19 pandemic. The adverse effects of the COVID − 19 pandemic on the socio-economic status and patient’s well-being may have additionally contributed to the low treatment adherence rates. The present findings relate to those published by authors in Zimbabwe’s Parirenyatwa hospital where radiotherapy uptake was reported in about 86% of the patients, with only 38% administered with chemotherapy [13]. In both settings, similar health system constraints are reported [13, 14, 25]. In another study done in a rural medical college in India, treatment completion rates for EBRT and Brachytherapy were also found to be considerably low (39%) due to poor accessibility of treatment facilities, patient age and socio-economic status [23]. Without proper adherence, the chances of patient survival are decreased, there are higher recurrence/treatment failure rates and increase of health care costs. The present findings and those reported in related studies call for the need for health system strengthening in resource-limited settings such as Uganda.

In this study, occupation, disease stage, distance from health facility, and missing a medical appointment were found to be significantly associated with treatment adherence in an initial bivariate analysis. However, no single patient factor was found to be independently associated with treatment adherence after multivariate analysis. In previous studies, treatment non- compliance was attributed to patient forgetfulness [15], long duration of therapy, complicated regimens, cost [15, 16], socio-economic status [26], disease stage [26–29], side effects of medications [15, 16, 28], distance to treatment facility [23, 28], missed medical appointment and poor understanding of treatment advantages [16], co-morbidities [28, 29], patient age [23, 26, 27], and having many children at home [23]. In the present study, the number of participants that were adherent to treatment (23 of 196) was too small to allow for the detection of possible associations. In some other studies, no differences in treatment adherence according to patient age and socio-economic status was found [28]. Additional studies are needed to confirm associations.

### Study limitations

There were some limitations in this study.

Except for patient factors, this study did not investigate the health system and other factors that could potentially influence treatment compliance.

## Conclusions

Treatment compliance was found in only 12% of the cohort participants. The association of patient factors with treatment compliance was not observed. Future studies may consider exploring health system and other factors to confirm the associations with treatment compliance.

## Data Availability

The clinical data sets used and /or analyzed during this study are available from the corresponding author upon request.

## Abbreviations

UCI: Uganda Cancer Institute
NCCN: National Comprehensive Cancer Network
EBRT: External beam radiation
HDR: High dose rate
SD: Standard deviation
AOR: Adjusted odds ratio
CI: Confidence interval
HIV: Human immunodeficiency virus
IRB: Institutional review board
